# P66Shc signals to age

**DOI:** 10.18632/aging.100057

**Published:** 2009-06-05

**Authors:** Mirella Trinei, Ina Berniakovich, Elena Beltrami, Enrica Migliaccio, Ambrogio Fassina, PierGiuseppe Pelicci, Marco Giorgio

**Affiliations:** ^1^ Congenia Srl, 20139 Milan, Italy; ^2^ Department of Experimental Oncology, European Institute of Oncology, 20139 Milan, Italy; ^3^ Department of Oncological and Surgical Sciences, Pathology Section, Cytophatology Unit, 35100 Padua, Italy

**Keywords:** Aging, Life span, degenerative disease, oxidative stress

## Abstract

Oxygen metabolism is thought to impact on aging through the formation of reactive oxygen species (ROS) that are supposed to damage biological molecules. The study of p66^Shc^, a crucial regulator of ROS level involved in aging dysfunction, suggests that the incidence of degenerative disease and longevity are determined by a specific signaling function of ROS other than their unspecific damaging property.

## What we can learn from longevity mutants

The reason why we age seems obvious: entropy increases. The reason why different species are differently affected by passing of equal time should be apparent as well: genetic and epigenetic variability. Organism modification with time has been mainly explained by the production of free radicals as well as by immunological theories of aging. However, what we still miss is a list of genes responsible for aging; the study of these genes would tell us what aging is.

*Senectus ipsa morbus est* (Old age is in itself a disease), ancient romans said. However, the incidence of disease decreases in the extreme elderly, when aging expression reaches its maximum, whereas progeric syndromes associate to disease. Therefore, it is not clear whether aging itself is a disease and how it would impact on life span in a protected environment.

Our contribution to this field arises from the study of p66^Shc^, the first protein identified whose deletion in mouse prolongs life span and protects from a variety of aging-associated diseases without showing apparent negative effects.

**P66**^**Shc**^** is a redox signaller **P66^Shc^ is a vertebrate protein. It is present in Xenophus, Botia Dario and mammals, while it is absent in Saccaromyces, Drosophila or Caenorhabditis [1]. P66^Shc^ is one of three isoforms encoded by the ShcA locus [2].

The other two isoforms, p46^Shc^ and p52^Shc^, with molecular weights of 46 and 52 KDa respectively, were first described as ‘adaptor' proteins that specifically bind to phosphorylated tyrosines on the cytoplasmic motif of growth factor receptors.  Upon growth factor stimulation, p52^Shc^/p46^Shc^ proteins are rapidly and efficiently tyrosine-phosphorylated by all the tyrosine kinase receptors tested in three major tyrosine residues, and recruit the Grb2-Sos complex on the plasma membrane [3]. In turn SOS, through its GEF activity, stimulates the conversion of the inactive Ras GDP into an active Ras GTP that subsequently activates the mitogen-activated protein kinase (MAPK) cascade. Recruitment of the Grb2/Sos complex by p52^Shc^/p46^Shc^ and membrane relocalization of Sos are events considered sufficient to induce Ras activation [3]. The hypothesis that Shc proteins are involved in the regulation of Ras is further supported by the finding that over-expression of p52^Shc^/p46^Shc^ increases  proliferative response and enhances MAP kinase and Fos activation upon stimulation with EGF, GM-CSF and PDGF [2,4,5]. Notably, the shortest isoforms of Shc appeared early in evolution since their orthologues have been found in flies and nematods [1].

At molecular level, p66^Shc^, p52^Shc^ and p46^Shc^ largely share the same amino acid sequence at the C-terminus including the Src homologous type two domain (SH2), phosphotyrosine binding domain (PTB) responsible for the binding to phosphorylated tyrosine, and a region highly enriched in glycine and proline residues named collagen homologous (CH1) since its homology with collagen protein [6]. The peculiarity of p66^Shc^ is an additional CH region (CH2) at its N-terminus [2,4].

Despite the high similarity p66^Shc^ functionally differentiates from the other ShcA isoforms. There is no indication that p66^Shc^ activates the Ras signaling pathway. Indeed, evidence for divergent regulation of p66^Shc^ versus p52^Shc^/p46^Shc^ immediately emerged from studies demonstrating that although p66^Shc^, like p52^Shc^ /p46^Shc^, is a target of receptor tyrosine kinases (EGFR, INSR, PDGFR) and binds the Grb2/SOS complex [4,7,8], p66^Shc^ over-expression, unlike that of p52^Shc^/p46^Shc^, has a negative effect on the Ras-MAPK-Fos pathway in response to EGF or cytokines in lymphocytes [4,9]. In fact, p66^Shc^ has been shown to exert an inhibitory effect on the Erk pathway, which is necessary for coordinated actin cytoskeleton polymerization [10], and normal IGF-1 responsiveness of the MEK/ERK pathway in myoblasts [11]. How p66^Shc^ exerts this negative effect is not clear. It was proposed that it acts by competing with p52^Shc^ for Grb2 binding, sequestering the Grb2/Sos complex and therefore terminating Ras signaling [11].

Finally, studies on p66^Shc^ knock down did not demonstrated any role for p66^Shc^ in growth factor response or Ras signaling whereas they revealed an unexpected function of p66^Shc^ in regulating intracellular redox balance and oxidative stress levels [12]. Indeed, compared to WT, the amount of reactive oxygen species (ROS) was shown to be decreased in p66^Shc^- depleted cultivated cells, as revealed by the reduced oxidation of ROS sensitive probes as well as by the reduced accumulation of endogenous markers of oxidative stress [9,13-17]. Likewise, p66^Shc^-/- mice show diminished levels of both systemic (isoprostane) and intracellular (nytrotyrosines, 8-oxo-dG) oxidative stress [14,18,19].

## Mechanisms of p66^ Shc^ - redox activity regulation

**Figure 1.**
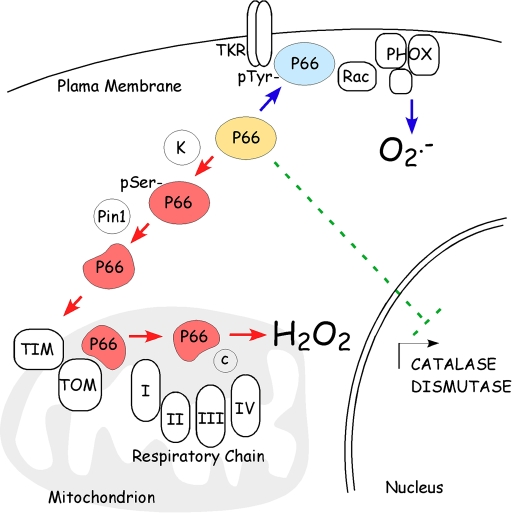
P66 ^Shc^ controls intracellular ROS metabolism. **at multiple sites. **P66^Shc^ (in blue) stimulates ROS production by plasma membrane oxidases through the association with membrane receptor and Rac activation of phagocitic oxidases. Upon phosphorylation and consequent Pin-1-mediated conformational changes, p66^Shc^ (in red) translocates, through the TIM/TOM mitochondrial import machinery, within the mitochondrial inter-membrane space where it oxidizes reduced cytochrome *c* and catalyzes the partial reduction of O_2_ to H_2_O_2_. Then, p66^Shc^ decreases the expression of ROS scavenging enzymes.

Basically, intracellular ROS levels can be increased by three main mechanisms: reducing ROS scavenging, increasing membrane oxidases activity, or by mitochondrial respiratory chain leakage.  P66^Shc^ has been reported to act through all of them. In fact, p66^Shc^ silencing by RNAi or gene targeting deletion was found to increase levels of superoxide dismutases and catalases in a variety of cells. In particular, p66^Shc^ appeared to decrease the expression of ROS scavenging enzymes through the inhibition of FOXO transcription factors [13] (Figure 1). In addition, p66^Shc^ has been proposed to mask the growth factor receptor bound protein Grb2 from Sos1, favoring the rac1-specific GEF activity of Sos1, rac1 activation and triggering of NADPH membrane oxidase ROS production [20] (Figure 1).

Finally, a fraction of p66^Shc^ has been observed within the mitochondrial inter-membrane space (IMS) [16]. Notably, electrochemical experiments demonstrated that the amino terminal portion of p66^Shc^ contains a redox active region able to mediate electron transfer from reduced cytochrome *c* to molecular oxygen, thus producing hydrogen peroxide (Figure 1).

As reported, all proteins of the mitochondrial inter-membrane space are synthesized in the cytosol and are then imported into the mitochondria [21]. Most of them do not contain any cleavable sequences and are targeted to IMS by as yet unidentified import signals.

The import of p66^shc^ into mitochondrial IMS is not still understood at a mechanistic level. However, a mechanism that depends on p66^Shc^ post-translational modifications, including serine phosphorylation by stress kinases like Jnk-1 and Pkc-B and prolilisomerization by Pin-1, has been described, which allows p66^Shc^ increase within the mitochondria during apoptosis [22]. A second level of activation of p66^Shc^ mitochondrial function is represented by the effective amount of p66^Shc^ within mitochondrial vesicles. In fact, mitochondrial p66^Shc^ has been observed to associate to a high molecular weight complex of about 670 KDa and to the mitochondrial chaperon mtHsp70 [23]. Notably, treatment of cells with pro-apoptotic stimuli such as UVC or H_2_O_2_ induces the dissociation of this complex and the consequent release of monomeric p66^Shc^,which is then free to react with cytochrome *c* [23].

Interestingly, p66^Shc^ half-life increases upon apoptotic stimulation in a p53-dependent way, thus linking the pro-apoptotic activity of p66^Shc^ to the p53 pathway [14].

## Function of p66^ Shc ^- oxidative signal

Regardless of how p66^Shc^ may shift the intracellular redox balance towards oxidation, it appears that p66^Shc^ specifically evolved to increase intracellular ROS levels. In this view, different functions have been assigned to p66^Shc^- produced ROS. Initially it was reported that H_2_O_2_ produced by p66^Shc^ within the mitochondria induces the opening of the mitochondrial permeability transition pore leading to swelling of the organelle  [16]. The consequent rupture of mitochondrial integrity then triggers the release of various proapoptotic mitochondrial factors, including cytochrome *c*, into the cytosol, where they activate the apoptotic cascade leading to cell death [23]. Indeed, p66^Shc^-/- cells have been demonstrated to be resistant to apoptosis induced by a variety of different signals, including ultraviolet radiation, staurosporine, growth factor deprivation, calcium ionophore, CD3-CD4 cross-linking and taxol [9,12,23]. Likewise, p66^Shc^-/- mice were found resistant to apoptosis induced by paraquat, hypercholesterolemia, ischemia, angiotensin II, carbon tetrachloride and ethanol [12,15,16,18]. Notably, p66^Shc^ deletion in mice was shown to improve resistance to hyperglycaemic damage in diabetic model of nephropathy and cardiovascular diseases due the reduction of apoptosis and cell loss [24,25].

**Figure 2.**
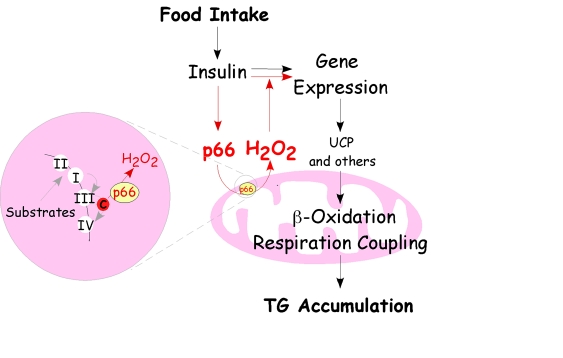
Regulatory circuit of p66 ^Shc^-mediated fat development. The scheme recapitulates the pathway of p66^Shc ^that drives mitochondrial H_2_O_2 _and its relationship with insulin receptor signaling leading to fat accumulation. Food intake determines energetic substrate availability and insulin stimulates intracellular transduction pathways that regulate gene transcription in order to favor triglyceride accumulation. P66^Shc^-mediated ROS production is directly boosted by insulin and in turn potentiates insulin receptor signaling, suppresses the expression of uncoupling proteins and beta oxidation enzymes leading to triglyceride accumulation.

Recently, another role for p66^Shc ^- mediated ROS has been described in the regulation of adipogenesis. In adipocytes, p66^Shc^ was demonstrated to be involved in insulin-induced gene expression regulation and triglyceride accumulation. In fat cells insulin induces serine 36 specific phosphorylation of p66^Shc^ thus stimulating p66^Shc^ ROS production, which, in turn, potentiates insulin transduction signaling. Indeed, mutants unable to translocate to the mitochondria and to produce H_2_O_2_ do not sustain insulin-dependent signaling and triglyceride accumulation when reintroduced in p66^Shc^-/- cells  [17]. Interestingly, some phosphatases inhibiting insulin signaling (e.g. PTEN) are inactivated by oxidation [26]. Thus, it appears that p66^Shc^-generated ROS play a crucial role in regulating insulin signaling and fat development, likely through the modulation of these redox-sensitive phosphatases. Indeed, p66^Shc^-/- mice are protected from diet-induced obesity, suggesting that this molecular pathway regulates diet-associated fat development [17]. But if p66^Shc^ is able to convert signals from the diet into variations of  the intracellular  redox balance,  affecting insulin sensitivity, critically, the process that triggers adipogenesis following food intake should stem from the integration of both intracellular (mitochondrial ROS production) and extracellular (circulating insulin) signals [17] (Figure 2).

Notably, p66^ Shc^ - produced H_2_O_2_ might control intracellular signaling events also in tissues other than fat. In particular, the response of myocytes and endothelial cells to glycaemia and ischemia, as well as the renewal control of breast stem cells upon hypoxia, has been linked to p66^Shc^- redox activity [24,27-29].

Therefore, p66^Shc^ behaves like an atypical signal transducer that tunes membrane receptor signaling or intracellular glucose/oxygen sensing via the regulation of intracellular re-dox balance.

## P66^Shc^ impacts on overall energy metabolism and aging

Was the p66^Shc ^gene conserved during mammals development, in spite of its deleterious effects on life-span and disease, because of p66^Shc^- mediated ROS signaling function in fat tissues?  P66^Shc^-/- mice have reduced body weight, due to reduced fat mass of both white and brown adipose tissues [17]. This leanness is not explainable by changes in food intake, intestinal absorption of nutrients or locomotor activity. Rather, it may reflect defective lipogenesis in adipocytes, as suggested by the reduced lipid accumulation of p66^Shc^-/- adipocytes transplanted into WT recipient mice [17]. However, this interpretation of the mechanisms leading to decreased fat mass in p66^Shc^-/- mice poses the question of how energy balance is maintained in the absence of p66^Shc^, and why energy storage is reduced. As p66^Shc^-/- mice showed increased basal body temperature and increased basal metabolic rate, this suggests that increased uncoupled respiration in the fat mitochondria of p66^Shc^-/- mice leads to increased energy expenditure, which contributes to resistance to body weight gain  [17].

Fat has a crucial role in the thermoregulation of mammals. It protects from body heat loss (thermoinsulation) and generates heat for the maintenance of body temperature when animals are exposed to cold (thermo-genesis). Notably, p66^Shc^-/- mice were found to be more sensitive to cold due to the reduced thermal insulation effect of fat pads [17]. Therefore, adaptation to cold as well as optimization of energy storage when food is available, both altered in the lean p66^Shc^-/- mice, have been proposed as possible evolutionary functions whose fitness pressure preserves the p66^Shc^ gene in mammals.

**Figure 3.**
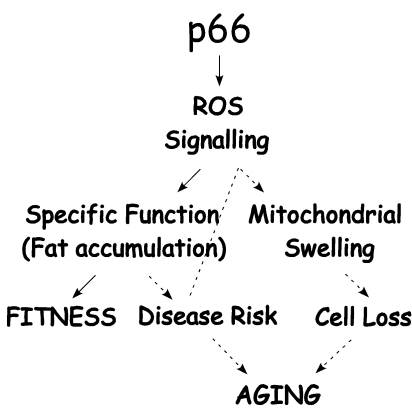
P66 ^Shc^/ROS signaling determines fitness and aging associated dysfunctions. P66^Shc^/ROS signals to specific functions that improve fitness whilst these same functions may increase disease risk chronically (such as obesity related disorders) and contribute to trigger p66^Shc^-mediated cell death. Then, increased disease risk and cell loss rate contribute to aging dysfunctions.

These findings of reduced adiposity in p66^Shc^-/- mice might have important implications for the effect of p66^Shc^ on lifespan. Aging is associated with a pathological trait, often associated with obesity (metabolic syndrome), which predisposes to diabetes and cardiovascular diseases [30-34]. In humans, these diseases strongly affect morbidity and mortality, especially among the elderly [30,35]. Oxidative stress has been implicated in a number of chronic disease states usually grouped under the umbrella of the metabolic syndrome [36-42], and it is thought to contribute to the aging process [43]. It has been hypothesized that the production of free radicals is dependent on metabolic rate [44], and that this may have an impact on the aging process. In p66^Shc^-/- mice, like in caloric restriction and FIRKO mice, fat deposits are moderately decreased [17,45], suggesting that reduced oxidative stress in p66^Shc^-/- mice might increase longevity through the direct effect of reduced adiposity.  Notably, p66^Shc^-/- mice are more resistant to diabetes and have reduced risk of atherosclerosis and cardiovascular damage upon HF-diet [18,25]. Therefore, the effect of p66^Shc^ on aging might be considered a sort of chronic decay like the metabolic syndrome progression, although the contribution of the metabolic syndrome to life span is still not clear (Figure 3).

The life-prolonging action of caloric restriction (CR) offers an excellent chance for investigating the connection between stress and aging. The anti-aging action of CR can be viewed as "nutritional stress," because the organism's reduced caloric intake seems to be a stimulatory metabolic response for survival. Thus, as an omnipotent intervention, CR provides a unique opportunity to probe the organism's ability to withstand age-related stress as a survival strategy. Recent geriatric research has provided sufficient experimental data supporting the anti-aging property of CR [46-48].

## What kills mammals is a "p66 ^Shc   ^syndrome"

Finally, the study of p66^Shc^ confirms that very close links exist between energetic metabolism, oxidative stress and aging. P66^Shc^ represents a clear example of an antagonistic pleiotropic function, which generates both beneficial and detrimental phenomena in an organism.

Darwin might say that aging expresses fitness (*senectus robur est*), at least as much as one is able to face illness. However, it remains unclear whether aging is also a disease or whether life span is regulated by energetic metabolism disorders that could eventually result in lethal effects or sub-pathological multiple dysfunctions.

In a series of WT and p66^Shc^-/- very old moribund mice, significant recurring cause of death were not identified. Indeed, it is known that in mice as in humans even accurate autopsy might often remain "blank", in the absence of masses, haemorrhages, abscesses or other evident septic conditions. Mice presented only sporadic terminal emphysema (mainly in WT mice), occasional lymphocytic pneumonia and very rare malignant tumors. On the other hand, it is impossible to rule out other causes of death, such as cardiac fibrillation or acute myocardial infarction, which score negative for morphological investigation (unpublished data).

In conclusion, whether aging determines life span through diseases or through the acceleration of a fatal physiological decline remains puzzling. It is expected that further, more intense investigations in the cause of death in mammals might contribute to the solution.

P66^Shc^ story suggests that necessary regulators of oxygen and energetic metabolism may be involved both in the onset of the acute phase of diseases and in the induction of aging related detrimental changes that ultimately kill the organism.
